# Investigation of Cellular and Molecular Responses to Pulsed Focused Ultrasound in a Mouse Model

**DOI:** 10.1371/journal.pone.0024730

**Published:** 2011-09-13

**Authors:** Scott R. Burks, Ali Ziadloo, Hilary A. Hancock, Aneeka Chaudhry, Dana D. Dean, Bobbi K. Lewis, Victor Frenkel, Joseph A. Frank

**Affiliations:** 1 Frank Laboratory, Radiology and Imaging Sciences, Clinical Center, National Institutes of Health, Bethesda, Maryland, United States of America; 2 Molecular Imaging Laboratory, Radiology and Imaging Sciences, Clinical Center, National Institutes of Health, Bethesda, Maryland, United States of America; 3 Imaging Sciences Training Program, Clinical Center and National Institute of Biomedical Imaging and Bioengineering, National Institutes of Health, Bethesda, Maryland, United States of America; 4 Intramural Research Program, National Institute of Biomedical Imaging and Bioengineering, National Institutes of Health, Bethesda, Maryland, United States of America; University of Pittsburgh, United States of America

## Abstract

Continuous focused ultrasound (cFUS) has been widely used for thermal ablation of tissues, relying on continuous exposures to generate temperatures necessary to induce coagulative necrosis. Pulsed FUS (pFUS) employs non-continuous exposures that lower the rate of energy deposition and allow cooling to occur between pulses, thereby minimizing thermal effects and emphasizing effects created by non-thermal mechanisms of FUS (i.e., acoustic radiation forces and acoustic cavitation). pFUS has shown promise for a variety of applications including drug and nanoparticle delivery; however, little is understood about the effects these exposures have on tissue, especially with regard to cellular pro-homing factors (growth factors, cytokines, and cell adhesion molecules). We examined changes in murine hamstring muscle following pFUS or cFUS and demonstrate that pFUS, unlike cFUS, has little effect on the histological integrity of muscle and does not induce cell death. Infiltration of macrophages was observed 3 and 8 days following pFUS or cFUS exposures. pFUS increased expression of several cytokines (e.g., IL-1α, IL-1β, TNFα, INFγ, MIP-1α, MCP-1, and GMCSF) creating a local cytokine gradient on days 0 and 1 post-pFUS that returns to baseline levels by day 3 post-pFUS. pFUS exposures induced upregulation of other signaling molecules (e.g., VEGF, FGF, PlGF, HGF, and SDF-1α) and cell adhesion molecules (e.g., ICAM-1 and VCAM-1) on muscle vasculature. The observed molecular changes in muscle following pFUS may be utilized to target cellular therapies by increasing homing to areas of pathology.

## Introduction

Focused ultrasound waves can be coupled with image guidance (e.g. magnetic resonance imaging (MRI)), to direct thermal and mechanical energy accurately deep within the body without causing demonstrable effects to the intervening soft-tissues or bone [1]. The current clinical use of focused ultrasound (FUS) exposures is to increase the temperature of targeted tissues (to 70–80°C) to generate coagulative necrosis and non-invasively treat uterine fibroids and prostate tumors [2]. Presently, FUS is being investigated in clinical trials for the treatment of other malignancies such as breast tumors and gliomas [2], [3]. Continuous FUS (cFUS) exposures (1–10 seconds) are typically accompanied by an inflammatory responses within the treated prostate tumor tissue [3]. Biermann et al [4] found mild and chronic inflammation in FUS-treated prostate tumors up to 180 days post FUS, but were not able to distinguish between cFUS-induced inflammation and tumor-associated or tumor-induced inflammation. As part of the inflammatory process, antigen-presenting cells (APC) (e.g. dendritic cells, macrophages, and B lymphocytes) have been observed at the periphery of cFUS-treated breast tumor lesions [5]. The APCs observed after cFUS treatment resulted in increased expression of T-cell-activating signals such as CD80 and CD86 suggesting that FUS treatment also stimulated an anti-tumor immune response. Hu et al. [6] also observed enhanced activity of cytotoxic lymphocytes and an increase in cells secreting tumor specific interferon-γ (INFγ) as a result of cFUS exposures in MC-38 colon adenocarcinoma tumors.

Whereas cFUS causes thermal ablation of tissue, shorter pulsed exposures (10–50 ms/sec) provide lower rates of energy deposition and allow cooling to occur between pulse intervals. Pulsed FUS (pFUS) exposures, despite utilizing relatively high intensities (1000–2000 Watts/cm^2^) minimize temperature elevations in tissue (∼4–5°C) [7], [8] and instead, emphasize the non-thermal effects of FUS (i.e. acoustic cavitation and acoustic radiation forces). These non-thermal effects have been shown to increase tissue permeability and enhance delivery of drugs and genes while inducing only minor and transient morphological changes within the treated region [9]. pFUS exposures to the brain result in transient disruption of the blood-brain-barrier (BBB), suggesting that this non-invasive tool can provide spatial and temporal control over delivery of therapeutics to brain tissue [2], [10]. These exposures may also be used for sonoporation, where transient pores are created by the collapse of cavitating bubbles to enhance local uptake of drugs and genes into individual cells [11]. Ultrasound-induced cavitation may also be employed to improve tissue plasminogen activator (tPA)-mediated thrombolysis [12]. Ultrasound exposures are also presently being developed where the energy (both thermal and nom-thermal) is used to deploy a variety of therapeutic agents from specially formulated carriers [13].

Relatively little is known about the cellular and molecular biological effects of pFUS exposures beyond the structural changes that result in vascular leakage. Cellular and molecular biology of tissues can be dramatically altered by mechanical force and stress through the process of mechanotransduction (i.e. biological activity in response to mechanical force) [14]. As the use of pFUS increases in clinical applications, there is a need to better understand the cellular and molecular consequences of depositing this form of energy in tissues and how to harness this non-invasive technique for novel therapies. Although pFUS is generally considered to be non-destructive, studies have provided some insight into the mechanism of action on sub-cellular and molecular levels (7–9). Following targeted pFUS exposures to muscle, *T*_2_-weighted MRI demonstrated the presence of edema that coincided with enhanced delivery of gadolinium (Gd) chelate-containing liposomes in the targeted regions [15]. pFUS of the muscle has been shown to create transient and reversible myobundle displacements or spreading (gaps) due to edema accompanied by possible disruption of the extracellular matrix (ECM) [16]. The changes in the muscle corresponded with improved distribution of fluorescently labeled nanoparticles injected directly into the tissue following the exposures. Histological examination revealed disruption of some capillaries and collagen in the ECM, but showed intact myofiber bundles. Trans-cranial pFUS exposures for the purpose of opening the BBB resulted in parenchymal enhancement on post-gadolinium chelate *T*_1_-weighed MRI [10]. pFUS performed in conjunction with intravenously-injected microbubbles produced an indiscrete lesion volume that was accompanied by transient extravasation of red blood cells (hemorrhage), inflammation, and macrophage infiltration into the targeted parenchyma [10]. Ischemic or apoptotic regions were not observed following pFUS, suggesting that the exposures were non-destructive to brain tissue. However, in these studies the molecular biological changes in the tissues following pFUS were not investigated.

The objectives of the current study were to characterize the cellular and molecular alterations in muscle following pFUS exposures with regard to local expression of cytokines, growth factors, and integrins. Fluorescently-labeled superparamagnetic iron oxide nanoparticles (FL-SPION) were intravenously injected in C3H mice to label splenic macrophages *in vivo* three days prior to pFUS or cFUS of the hamstring muscle. Following FUS, animals underwent *T*_2_- and *T*_2_*-weighted MRI to detect the presence of edema and FL-SPION-labeled cells, respectively. Mice were euthanized at specific time points for histological, cellular, and molecular analyses of the tissue. *T*_2_-weighted MRI revealed the presence of significant edema in cFUS-treated muscle that persisted through 8 days post-FUS that was not observed in pFUS-treated muscle. *T*_2_*-weighted MRI revealed regions of low signal intensity (hypointense voxels) up to 8 days after both cFUS and pFUS treatment that corresponded to infiltration of SPION-labeled macrophages in and around muscle bundles when examined by fluorescence microscopy and Prussian blue staining. Unlike cFUS, pFUS induced little or no apoptosis in treated muscle. Importantly, early elevations in cytokine expression were detected in both cFUS- and pFUS-treated muscle (0 and 1 days post-FUS) compared to muscle in the untreated contralateral leg. In general, cytokine levels in the treated muscle returned to contralateral muscle levels by 3 days post-FUS. In pFUS-treated muscle, acute and transient cytokine expression included; interleukin 1 (IL-1α, IL-1β), tumor necrosis factor (TNFα), monocyte chemotactic protein (MCP-1), macrophage inflammatory protein (MIP-1α) in treated muscle. Accompanying the local upregulation of cytokines in pFUS-treated muscle were also increases in growth factors [e.g. vascular endothelial growth factor (VEGF), fibroblast growth factor (FGF), stromal cell-derived factor (SDF-1α)] and increased expression of the cell adhesion molecules intercellular adhesion molecule-1 (ICAM-1) and vascular cell adhesion molecule-1 (VCAM-1) on the muscle vasculature.

## Results

### Labeling of macrophages with FL-SPION *in vivo*

**Figure 1:**
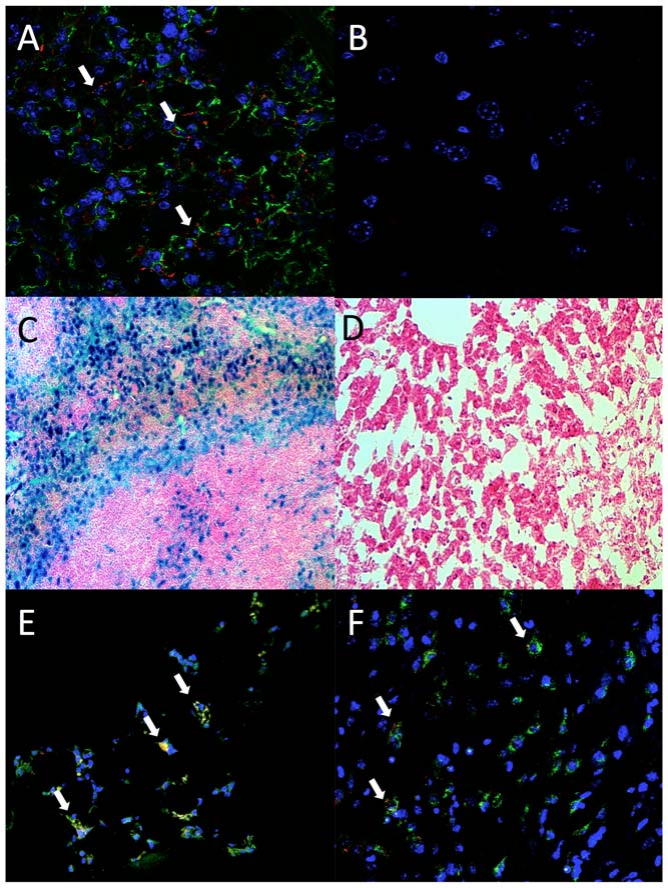
Histological detection of FL-SPION in the liver and spleen 3 days post-injection with FL-SPION, and in FUS-treated muscle 6 days post-injection of FL-SPION and 3 days post-FUS. **A** Confocal microscopy shows FL-SPION (red) in the spleen are localized with macrophage-specific F4/80 IHC staining (green). **B** FL-SPION are not as abundant in liver **C** Prussian Blue staining confirms FL-SPION are predominately taken up in the spleen rather than the liver (D). FL-SPION-labeled macrophages are detected in leg muscle after receiving cFUS (E) and pFUS (F) exposures. Arrows indicate macrophages that are positive for both F4/80 and FL-SPION.

FL-SPION were intravenously administered to mice 3 days prior FUS. On the FUS treatment day, control mice that did not receive FUS were euthanized and tissue sections from spleens and livers were examined for the presence of labeled macrophages by confocal microscopy and Prussian blue staining (Figure 1A–D). FL-SPIONs were detected predominately in the spleen. FL-SPION fluorescence appeared in regions that were positive for immunohistochemical (IHC) staining of the macrophage-specific marker F4/80 (Figure 1A). By 3 days post-FUS FL-SPIONs were visible by fluorescence microscopy and associated with F4/80 fluorescence in muscle tissue that received either pFUS or cFUS (Figure 1E and F). Threshold fluorescence intensity values were obtained by imaging controls of each tissue type from mice that did not receive FL-SPION and were immunostained without the F4/80 primary antibody.

### Monitoring FL-SPION-labeled macrophage infiltration by MRI

**Figure 2:**
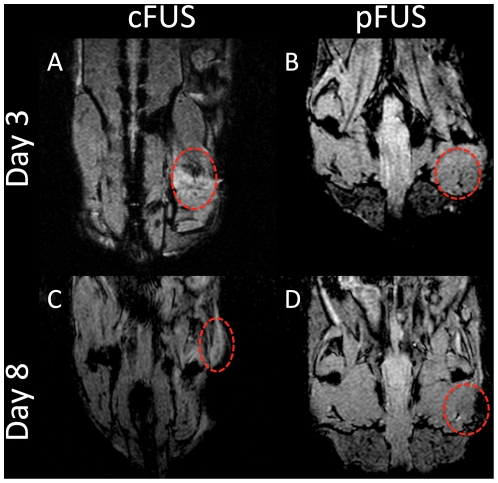
Evaluation of FL-SPION-labeled macrophage migration to FUS-treated muscle tissue by MRI. *T*_2_*-weighted MR images were obtained at 3T after 3 (**A** and **B**) and 8 (**C** and **D**) days post-FUS. Imaging reveals hypointense voxels in regions of the right leg that were treated with cFUS (**A** and **C**) or pFUS (**B** and **D**) exposures.

**Figure 3:**
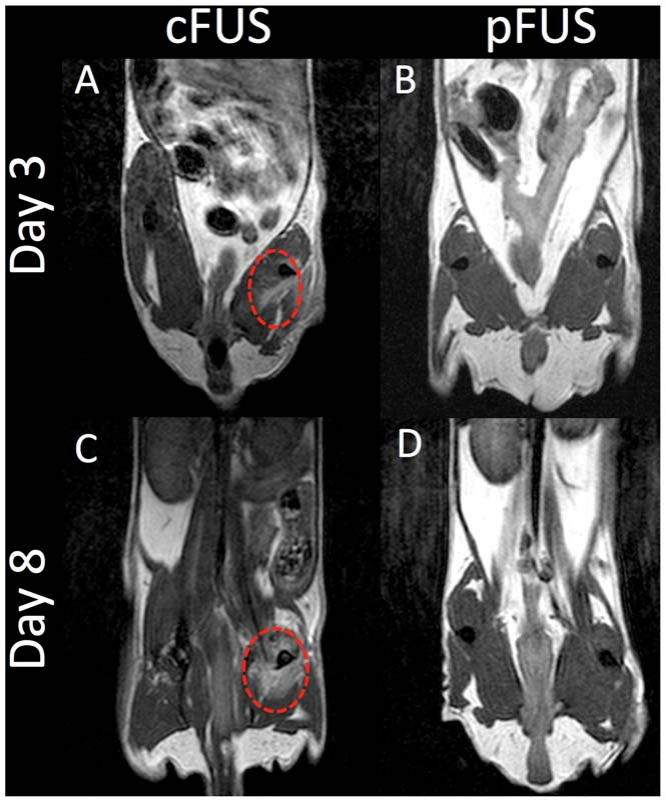
Evaluation of FUS-treated muscle tissue by *T*_2_-weighted MRI. *T*_2_-weighted MR images were obtained at 3T after 3 (**A** and **B**) and 8 (**C** and **D**) days post-FUS. Imaging reveals persistent edema on days 3 and 8 in regions of the right leg that were treated with cFUS exposures (**A** and **C**) but not pFUS exposures (**B** and **D**).

Labeling macrophages with FL-SPION allowed non-invasive monitoring of macrophage infiltration *in vivo* by clinically relevant MRI at 3 Tesla. *T*_2_*-weighted MR images of mice that received FL-SPION followed by either cFUS or pFUS to the leg were acquired 3 and 8 days after treatment with FUS. Figure 2 shows representative MR images demonstrating hypointense voxels in the region of the leg that received FUS exposures that was not detected in the contralateral untreated legs. Hypointense voxels were detected after both cFUS or pFUS treatment at 3 days post-FUS and persisted through 8 days post-FUS. Qualitatively, hypointense voxels were clearly observed in cFUS-treated animals on days 3 and 8 (Figure 2A and C), and pFUS-treated animals on day 3 (Figure 2B). pFUS-treated animals on day 8 demonstrated a more homogenous distribution of poorly-contrasted hypointensity through the treated area (Figure 2D). *T*_2_-weighted images at the same time points show evidence of edema as hyperintense voxels in the treated legs of mice receiving cFUS, however, edema was not detected in pFUS-treated mice (Figure 3).

### Histological characterization of FUS-treated muscle and quantification of macrophage infiltration

**Figure 4:**
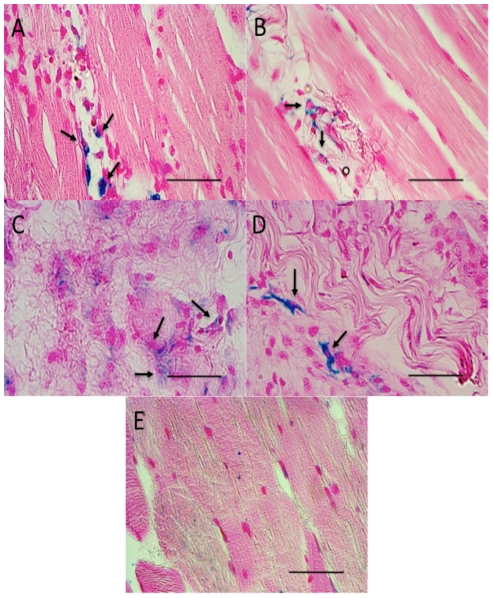
Prussian blue staining of FL-SPION-labeled macrophages in muscle tissue following FUS exposures. Muscle following pFUS on days 3 (**A**) and 8 (**B**) post-treatment. pFUS-treated tissue shows well preserved cytoarchitecture with increased gaps between muscle fiber bundles and infiltration of macrophages (arrows). Tissue exposed to cFUS is shown 3 (**C**) and 8 (**D**) days after. Large amounts of necrosis and macrophage infiltration are seen with no discernable histological integrity through the treatment volume. Control untreated muscle is shown in (E). Scale bars represent 50 µm.

Prussian blue staining was performed on FUS-treated muscle sections to detect FL-SPION-labeled macrophages 3 and 8 days post-FUS (Figure 4). Animals receiving ablative cFUS exposures were found to be necrotic with highly disorganized cytoarchitecture compared to the contralateral limb (Figure 4C and D). In mice that received pFUS, the morphological integrity was highly preserved throughout the treatment volume (Figure 4A and B). pFUS-treated muscle exhibited less necrosis than cFUS-treated tissue at both days 3 and 8. Extensive hemorrhage was frequently observed in all mice treated with cFUS. In contrast, only rare areas of hemorrhage were noted following pFUS and evidence of hemorrhage was not observed in all pFUS-treated mice (Figure S1). These findings are consistent with a previous report in which intact muscle fibers were observed to occur with increased gaps between myofibrils [16]. Histological findings were unchanged between days 3 and 8 for both pFUS- and cFUS-treated animals.

**Figure 5:**
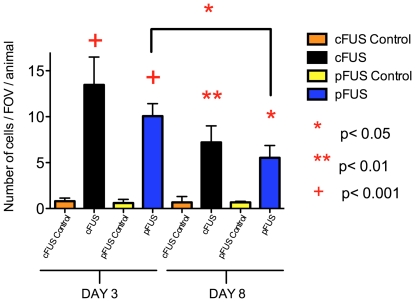
Quantification of macrophage infiltration into muscle following cFUS or pFUS. Muscle tissue treated with cFUS or pFUS showed a greater number of Prussian blue positive macrophages than control tissue on both days 3 and 8 post-FUS (5 FOV/animal, n = 3). Statistically similar numbers of macrophages were observed in pFUS- and cFUS-treated tissue on day 3. A significant decrease (p<0.05) in macrophages is observed between days 3 and 8 in pFUS-treated tissue, but not in cFUS-treated tissue.

Macrophage infiltration into muscle was quantified by counting Prussian blue-positive cells in the treatment volume. Five fields-of-view (FOV) were analyzed using a 40× objective to view similar regions of the treatment volume from 3 animals per treatment group. FUS treated muscles were compared to the untreated contralateral leg in each mouse (Figure 5) (*F*_7,23_ = 36.34, *p*<0.0001). The presence of macrophages was greater in both cFUS- and pFUS-treated groups at days 3 and 8 when compared to controls of the same day. Macrophage infiltration was not statistically different between pFUS or cFUS treatment on day 3 (p>0.05). The number of observed macrophages in pFUS-treated animals did decrease between days 3 and 8 (p<0.05). There was a statistically insignificant trend for macrophage infiltration to decrease between days 3 and 8 in mice receiving cFUS (p>0.05).

### Effects of cFUS and pFUS on apoptosis

**Figure 6:**
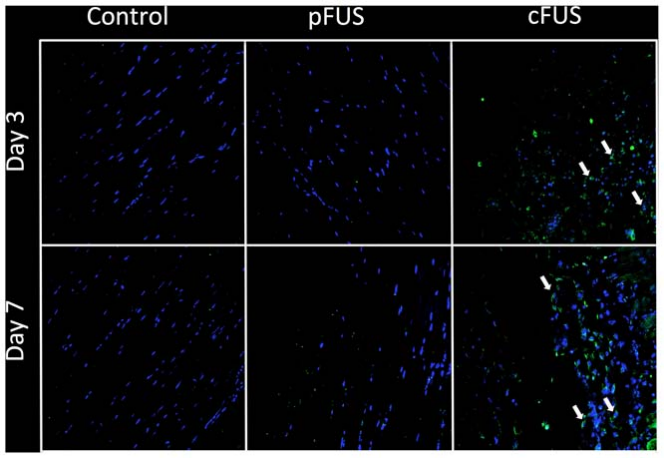
Effect of FUS on apoptosis. Blue represents DAPI-stained nuclei and green represents TUNEL-positive nuclei. No apoptotic nuclei were observed on days 3 or 8 following pFUS or in untreated control muscle at the same time points. However, several apoptotic nuclei were observed near the margin of the cFUS treatment volume on both days 3 and 8 post-cFUS.

To further investigate whether pFUS exposures were destructive to tissue, apoptotic nuclei were detected using a fluorescein-based TUNEL assay. Muscle tissue sections from pFUS- and cFUS-treated animals were stained for TUNEL-positive nuclei and compared to untreated contralateral legs. Apoptotic nuclei were detected near the margins of the treatment volume in animals receiving cFUS on both days 3 and 8 following treatment (Figure 6) and were not observed in pFUS-treated mice. Sections from pFUS-treated mice resembled those from control tissue.

### Upregulation of cytokines, growth factors, and cell adhesion molecules

**Figure 7:**
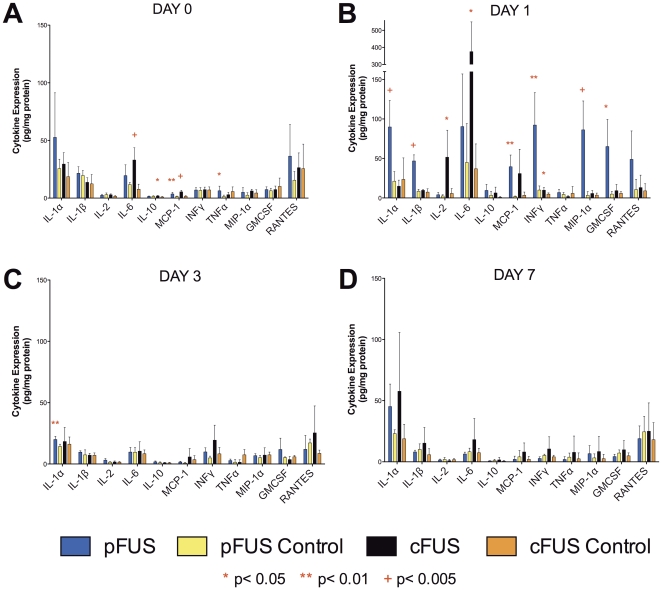
Expression of proinflammatory cytokines in muscle following cFUS or pFUS. Levels of each cytokine in muscle treated with cFUS or pFUS (n = 5) was compared to the control tissue of the same day. Most cytokine elevations were short-lived and returned to control levels by day 3.

To investigate the molecular responses that accompanied the deposition of FUS energy (primarily thermal energy for cFUS and mechanical energy for pFUS), we examined levels of signaling molecules including cytokines, growth factors, and cell adhesion molecules. For cytokine levels, homogenized muscle tissue was analyzed using an ELISA-based cytokine array. Tissues from both pFUS- and cFUS-treated animals (n = 5) were analyzed for levels of cytokines on days 0, 1, 3, and 7 following FUS exposures. To assess local increases in cytokine levels, those in treated muscle were compared to control muscle from the contralateral leg on the same day. The data are summarized in Figure 7 and Figure S2 (see Table S1 for statistical values). Two-way ANOVAs performed for each cytokine revealed that both cFUS- and pFUS-treated muscle tissue exhibited significant increases of several cytokines on days 0 and 1 following treatment, and in general, declined to control levels by 3 days post-treatment.

The panel of cytokine expression differed somewhat between cFUS and pFUS treatment. pFUS upregulated local expression of the following cytokines on days 0 or 1 after treatment: IL-1α, IL-1β, MCP-1, INFγ, MIP-1α, granulocyte-macrophage colony-stimulating factor (GMCSF), and RANTES. cFUS upregulated expression IL-2, IL-6, IL-10, MCP-1, and INFγ during the same time period. Cytokine levels in treated tissue were identical to those in the control tissue by day 3 after FUS treatment with the exception of a modest elevation in IL-1α being observed 3 days after pFUS. In pFUS-treated tissue, IL-4, IL-5, IL-6, IL-10, and IL-17 were greater in treated tissue on days 0 and 1 than at days 3 or 7 (Figure S1). However, levels of these cytokines in treated tissue on days 0 and 1 were not different than contralateral muscle levels on days 0 and 1. This suggests the possibility of a systemic release of these interleukins in the window immediately following pFUS.

**Figure 8:**
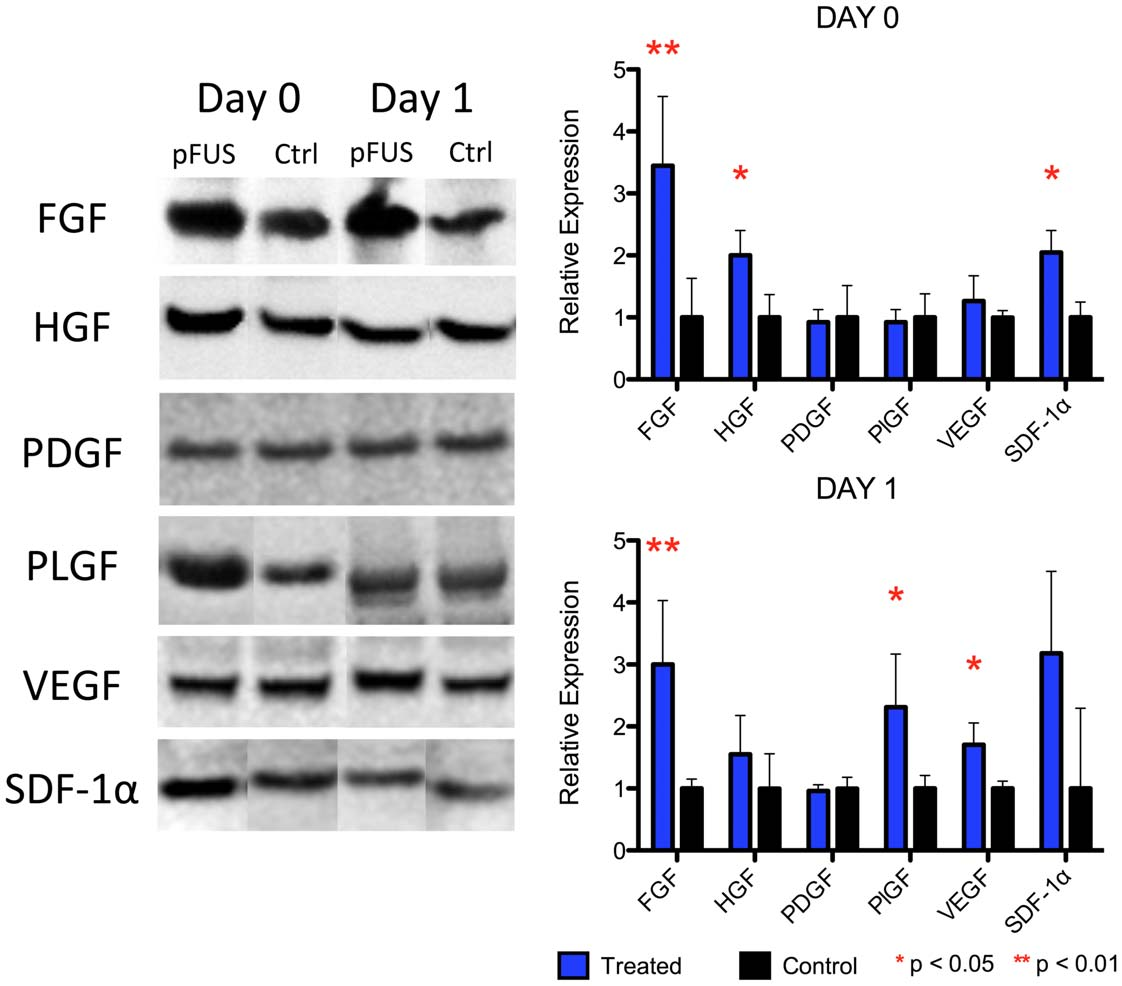
Expression of growth factors on days 0 and 1 following pFUS treatment. Western blotting was used to compare growth factor expression in pFUS-treated muscle compared to muscle from the untreated contralateral leg (n = 5).

We examined the local expression levels of chemotactic growth factors in pFUS-treated tissue that are associated with inflammation, cell homing, and tissue regeneration. We examined expression of these factors on days 0 and 1 post-treatment (n = 5)—during the window when cytokine levels were elevated (Figure 8, Table S2). Western blotting showed significant increases in expression of VEGF, FGF, SDF-1α, hepatocyte growth factor (HGF), and placental growth factor (PLGF) in pFUS-treated tissue within 24 hr following treatment when compared to the contralateral leg. Of note, PDGF levels were not different in the pFUS treated versus contralateral legs.

**Figure 9:**
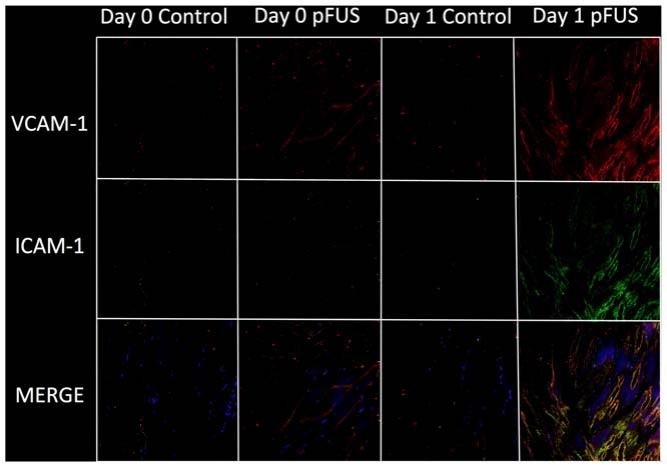
Expression of ICAM-1 and VCAM-1 in muscle tissue on days 0 and 1 following pFUS. Blue represents DAPI stained nuclei, green represents ICAM-1, red represents VCAM-1, and yellow represents merged ICAM-1 and VCAM-1. Compared to control, there is slight increase of VCAM-1 on day 0 and dramatic increase of both ICAM-1 and VCAM-1 on day 1 post-pFUS.

Another component of cellular infiltration into tissue is the expression of signaling/adhesion molecules such as ICAM-1 and VCAM-1, which are overexpressed on activated endothelial cells. Dual fluorescence-immunostaining for ICAM-1 and VCAM-1 was performed on tissue sections on days 0 and 1 following pFUS treatment (Figure 9). Compared to contralateral control muscle, the fluorescence images show pFUS treatment results in a modest increase of VCAM along muscle fiber bundles on day 0, and dramatically increased expression of both VCAM and ICAM in vessels and muscle fibers on day 1.

## Discussion

The major finding of this study is that pFUS exposures induced an array of molecular biological changes in muscle tissue and infiltration of macrophages without detectable destruction to exposed tissue. By pre-labeling splenic macrophages with FL-SPION, we were able to non-invasively track the infiltration of macrophages to the exposure site by histology and *T*_2_*-weighted MRI. pFUS exposures induce a short-lived molecular response in treated muscle including the upregulation of cytokines, growth factors, and adhesion molecules.

Previous characterizations of cellular and molecular responses of pFUS exposures have been incomplete and not specifically focused on factors regulating cellular homing [10], [15], [16], [17]. Histologically, pFUS exposures in the brain were shown to produce indiscrete lesions [10], [17]. Post FUS, limited extravasation of red blood cells and infiltration of macrophages were observed persisting up to 4 weeks, and did not appear do induce significant amounts of neuronal damage, necrosis, or apoptosis [10]. pFUS exposures to the brain have been shown to increase BBB permeability through the disruption of endothelial tight junctions [17]. pFUS exposures to the muscle were shown to transiently increase tissue permeability through enlargement of gaps between muscle fiber bundles and improve convective transport of locally-injected nanoparticles [16]. Our study agrees with previous findings in muscle that suggest pFUS, unlike cFUS, can be applied to tissue without observed destruction to the tissue [9], [16]. In the current study, the hamstring muscle did not exhibit deleterious effects in response to pFUS, whereas major myofiber disorganization, necrosis, and apoptosis were observed with cFUS. pFUS resulted in enlarged gaps between muscle fibers with some small amount of hemorrhage through 8 days of this study. Macrophage infiltration following pFUS was similar to that cFUS treatment on day 3, but significantly reduced by day 8 in pFUS-treated tissue compared to cFUS-treated tissue. By day 3 post-pFUS, edema was not detectable by *T_2_*-weighted MRI unlike cFUS, where persistent edema was detected through day 8. pFUS treatment did not induce apoptosis that was observed in cFUS-treated tissue, especially near the margins of the treatment volume.

Relatively little is known about the biochemical and molecular biological changes following pFUS. One previous study conducted a gene array analysis on muscle treated with pFUS and revealed upregulation in a variety of genes, including some involved in inflammation and cell homing processes [18]. The pFUS used in that study [18] deposited approximately 65 times more total energy to tissue than exposures described in the current study and elsewhere [16]. Furthermore, the authors of that study [18] observed significant necrosis and disorganization of muscle tissue. Molecular responses to lower-power pFUS exposures, where tissue destruction was not observed, have heretofore not been described.

The mechanical disruptions of pFUS exposure triggers an acute and short-lived cascade of cytokines and growth factors that are involved in macrophage infiltration, wound healing, and anti-inflammatory responses. Significant interactions occur between many of the upregulated cytokines in skeletal muscle [For Review see [19]]. For example, TNFα and INFγ upregulate expression of ICAM and VCAM [20], [21] while TNFα can also stimulate local release of IL-1β, which in turn, also increases ICAM and VCAM [22] expression on the surfaces of endothelial cells. IL-1β also activates endothelial cells to secrete GMCSF [23] whereas it has been shown that MIP-1α recruits circulating monocytes to tissue [24]. MCP-1 is involved in recruitment and activation of macrophages [25]. In response to pFUS exposures, several growth factors are also expressed to facilitate homing of immune cells and tissue regeneration. HGF and FGF enhance myoblast proliferation while HGF also increases myoblast migration into the injured area [26], [27]. VEGF and PLGF stimulate angiogenesis [28], [29] along with SDF-1α [30], and can recruit muscle progenitor cells from bone marrow [31]. In the current study ICAM and VCAM were upregulated in the pFUS-treated muscle suggesting that mechanotransduction may have induced these changes as well as the cytokine expression, which result in the movement of macrophages and other cells into interstitial spaces [32].

Mechanotransduction from pFUS exposures stimulates vigorous molecular responses that may be associated with inflammation, tissue repair, and cell homing without significant tissue destruction, and potentially may have great clinical value. Low-intensity (i.e., therapeutic) ultrasound using microbubble contrast agents has been shown effective in accelerating hyperemia blood flow in skeletal muscle after ischemic events with some evidence it also promotes remodeling of the microvasculature [33]. Inducing mechanotransduction in a non-destructive way may be a reliable technique to target homing of cell-based therapies. For example, bone marrow stromal cells or mesenchymal stem cells (BMSC) are capable of either actively or passively homing to sites of inflammation where local cytokine gradients are present, but is often inefficient with regard to the number of cells reaching these regions [34], [35]. pFUS exposures essentially provide a way to modulate, both spatially and temporally, the expression of pro-homing factors in tissues. Energy from pFUS can be accurately deposited deep in the body, is safe, and a readily translatable technology to the clinic. An additional advantage of this treatment modality (cFUS or pFUS) is the relatively small size of the focal zone (typically 1 mm×1 mm×10 mm), where acoustic energy is concentrated. This renders FUS highly specific in regards to targeting. For example, treatment of prostate tumors can currently be carried out without destructive effects in sensitive structures such as the neurovascular bundle [36].

The results of the present study suggest that pFUS exposures may be an ideal way to target homing of stem cells at a desired time and to a desired location by increasing local expression of molecular cues. The acute release of cytokines and growth factors from muscle following pFUS indicates that molecular changes occur as a result of non-thermal radiation forces. This, coupled with the apparently non-destructive manner in which energy is deposited could potentially be used to enhance tropism of cells to target tissue. We have observed in a preliminary study, increased BMSC migration to kidneys as a result of pFUS treatment compared to the untreated contralateral kidney (unpublished results). Several aspects of this phenomenon require further investigation including determining if pFUS can stimulate additional stem cell homing to target tissue during active inflammation or pathology. Furthermore, molecular characterization in other tissues following pFUS is needed. Specifically, we seek to understand if multiple pFUS exposures to the same site can reestablish pro-homing factor gradients within tissue once they have decayed back to baseline following initial exposures or after acute inflammation from pathology has subsided. Especially of importance, is whether pFUS exposures can establish chemokine gradients in pathological models after the acute inflammatory window has closed, which potentially frees cellular therapeutics from the necessity to be administered during this early time period. These investigations are ongoing in the laboratory.

## Materials and Methods

### Animals

All animal procedures were done in accordance the Animal Care and Use Committee of the Clinical Center at our institution. Female C3H mice (∼16 weeks of age) were housed with free access to food and water and were allowed to reach a weight of >28 g before FUS exposures. Mice were administered a tail vein injection of Molday ION Rhodamine-B SPION (8 mg/kg bw, BioPAL, Worcester, MA) 72 h prior to FUS treatment, and hair on both legs was removed with depilatory cream 24 h prior.

### FUS

A single treatment of FUS was administered to the right legs of mice using a modified Sonoblate 500 system (Focus Surgery, Indianapolis, IN) described previously [16]. The probe was comprised of both a spherical, concave therapeutic transducer (diameter, 5 cm; focal length, 4 cm; operating frequency, 1 MHz; max power output, 120 W) and a collinear imaging transducer (aperture, 8 mm; operating frequency, 10 MHz). During the procedure mice were anesthetized with isoflurane (2.5% in O_2_) and placed in a restrainer with their legs submerged in degassed H_2_O maintained at 37°C. Mice were positioned under imaging-ultrasound guidance such that therapeutic exposures were aimed at the right hamstring muscle. Therapeutic FUS was performed across the hamstring with a raster pattern of a 2×3 matrix in the X–Y plane (i.e. perpendicular to the direction of ultrasound propagation) with elemental spacing of 2 mm. For pulsed exposures, each of the 6 foci received 100 cycles of FUS using the following exposure parameters: acoustic power, 40 W; pulse repetition frequency, 1 Hz; duty cycle, 5% (50 ms “on” and 950 ms “off”). The total exposure time per raster point was 1.67 min. cFUS exposures were set up in an identical manner, but each focal point received a single pulse lasting 4 s at 100 W. The spatial average, temporal peak intensity (I_SATP_) of pFUS and cFUS exposures was 2660 W/cm^2^ and 6650 W/cm^2^, respectively. The spatial average, temporal average intensity (I_SATA_) was the same as the I_SATP_ for the cFUS exposures, but was 133 W/cm^2^ for the pFUS exposures. The total energy deposited by pFUS and cFUS exposures was 1.33×10^4^ J and 2.67×10^4^ J, respectively.

The parameters of the pFUS exposures were selected on the following basis: they have been shown to enhance the delivery of nanoparticles to muscle while minimizing the thermal effects of FUS (generating temperature changes between 2–4°C). cFUS exposures were designed to generate similar effects observed with tumor ablation in clinical treatments (9). Mice were euthanized at 4 h, 1, 3, and 7 or 8 days after FUS treatment. The vasculature of the mouse was perfused with 0.9% saline or 4% paraformaldehyde in PBS (pH 7.4) through the left ventricle. A portion of the right atrium was excised the mouse was perfused until clear perfusate emerged from the right atrium. Left and right hamstrings, spleens, and livers were collected and used for molecular and histological analyses.

### Immunohistochemistry

Tissue was immersed in 4% paraformaldehyde in PBS (pH 7.4) overnight at 4°C. Tissue was embedded in paraffin and sectioned at a thickness of ∼10 µm and mounted onto positively-charged slides. Paraffin was removed in xylene and tissue was rehydrated in graded ethanol concentrations. Tissues were washed extensively with PBS containing 0.05% Tween-20 (TPBS). Heat induced epitope retrieval (HIER) was performed by microwaving tissue for 4 min in HIER-citrate buffer. Tissue was allowed to cool and then was treated with SuperBlock (Pierce Biotechnology, Rockford, IL) for 5 min and rinsed with TPBS. Tissues were incubated with primary at a 1∶50 dilution in TPBS overnight at 4°C. Primary antibodies against ICAM and F4/80 were rat anti-mouse IgG and antibodies against VCAM were rabbit anti-mouse IgG. Tissue was rinsed extensively in TPBS and then incubated in the dark for 2 hr at room temperature with an AlexaFluor 488-conjugated goat anti-rat IgG or AlexaFluor 546-conjugated goat anti-rabbit IgG as a secondary antibody. Tissues were again washed thoroughly with TPBS and #1-glass coverslips were affixed using ProLong Gold antifade reagent with DAPI (Invitrogen).

### Confocal Microscopy

Muscle sections were examined using an upright laser scanning confocal microscope (series 710, Zeiss, Oberkochen, Germany) using Plan-Apochromat objectives (20× air, N.A. = 0.8; 63× oil-immersion, N.A. = 1.4). Illumination was provided by an argon-ion (Lasos, Jena, Germany), diode, and diode-pumped solid-state lasers (Roithner Lasertechnik, Vienna, Austria). Excitation for DAPI, Fluorescein/AlexaFluor488, and Rhodamine-B/AlexFluor546 was performed using laser lines at 405 nm, 488 nm, and 561 nm, respectively. Fluorescence emission was filtered using a short-pass (495 nm), band-pass (494–542 nm), and long-pass (566 nm) filters appropriate for each fluorophore. Fluorescence images were acquired sequentially to minimize cross-talk.

### MRI

Mice were imaged on a 3T clinical scanner (Acheva, Philips Healthcare, Andover, MA) using a 4 cm solenoid receive-only coil (Philips Research Laboratories, Hamburg, Germany). *T*_2_*-weighted images were acquired with the following instrumental settings: repetition time (TR), 430 ms; echo time (TE), 15 ms; flip angle, 30°; number of signal acquisitions (NSA), 4. *T*_2_-weighted images were acquired during the same imaging sessions. Dual-echo, turbo spin echo (TSE) images were acquired with the following instrumental settings: TR, 2128 ms; TE, 10 and 50 ms; echo train length (ETL), 6; number of averages = 3. Images were obtained with a 5 cm field-of-view and 0.5 mm slice thickness. Images were reconstructed using 512 × 512 pixels.

### Prussian Blue Staining

Fixed tissue sections were deparaffinized and rehydrated as described above. Prussian Blue staining was done by incubation in 10% potassium ferrocyanide and 10% hydrochloric acid for 30 min. Slides were then washed extensively in deionized water and counterstained with Nuclear Fast Red (Scytek, Logan, UT) for 3 min. Slides were dehydrated in graded ethanol and xylene and #1-glass coverslips were affixed using Permount mounting medium. Slides were examined on an Axioplan Imaging II microscope (Zeiss) illuminated with an X-cite mercury light source (model 120Q, Lumen Dynamics, Mississauga, ON) using 20× air (N.A. = 0.75) and 40× oil immersion (N.A. = 1.4) objectives.

### TUNEL Staining

Apoptotic cells were detected in deparaffinized tissue sections using a fluorescein-based *in situ* cell death detection kit (Roche Applied Science, Indianapolis, IN) according to manufacturer's protocols. Sections were imaged by confocal microscopy for the presence of fluorescein-positive nuclei.

### Cytokine Array Analysis

Harvested hamstring muscle was homogenized in cell lysis buffer (Roche Applied Science) containing protease inhibitor cocktail (Santa Cruz Biotechnology, Santa Cruz, CA) at 4°C. Samples were centrifuged at 15,000 rpm for 20 min at 4°C. The supernatant of each sample was collected and used for analysis. Total protein content of each sample was determined using a bicinchoninic acid assay (Pierce Biotechnology). Cytokine levels for pFUS-treated and untreated tissue were measured using a 16-plex Mouse Cytokine Screen (Quansys Biosciences, Logan, UT). The assay was performed according to manufacturer's protocols with each sample at a protein concentration of 3 mg/mL. Luminescence was measured on an ImageStation 4000R Pro (Carestream Molecular Imaging, Woodbridge, CT) using an exposure time of 5 min.

### Western Blotting

Protein samples (50 µg) were separated by sodium dodecyl sulfate-polyacrylamide gel electrophoresis (SDS-PAGE) under reducing conditions on Novex Bis-Tris gels (4–12% acrylamide, Invitrogen) and then transferred to polyvinylidene fluoride (PVDF) membranes. Membranes were blocked using 5% bovine serum albumin (BSA) in TBS+0.05% Tween-20 (TTBS) at room temperature for 1 hr. Membranes were hybridized with rabbit anti-mouse IgG primary antibodies against vascular endothelial growth factor (VEGF), fibroblast growth factor (FGF), platelet derived growth factor BB (PDGF-BB), placental growth factor (PLGF), hepatocyte growth factor (HGF), and stromal cell derived growth factor 1α (SDF-1α) overnight at 4°C in TTBS containing 5% BSA. All primary antibodies were from Abcam (Cambridge, MA) and were used at a 1∶1000-fold dilution from manufacturer's stocks. Hybridization with a secondary antibody was done for 1 hr at room temperature using a horseradish peroxidase (HRP)-conjugated, donkey anti-rabbit-IgG antibodies at a 1∶5000 dilution from manufacturer's stock (GE Healthcare, Little Chalfont, UK). Blots were developed by incubation with enhanced chemiluminescence reagents (Invitrogen) for 2 min at room temperature and imaged with an ImageStation 4000R Pro (Carestream Molecular Imaging) with exposure times ranging from 1–10 min. Loading controls were performed with Ponceau-S staining of the membranes.

### Data Analyses

Qualitative data analyses were performed by researchers in a blinded fashion. Quantiative values are presented as the mean±S.D. Comparison of means was done using a two-way analysis of variance (ANOVA) followed by Bonferroni's post-test or a Student's t-test with a Bonferroni correction using Prism software (v. 5.0, GraphPad Software, La Jolla, CA). A *p*-value<0.05 was considered significant.

## Supporting Information

Figure S1 Prussian blue staining of FL-SPION-labeled macrophages in muscle tissue following FUS exposures. Muscle after cFUS (A) and pFUS (B) 3 days post-treatment. Using Nuclear Fast Red as a counterstain, anuclear red blood cells are indicated by arrows and appear as small disc-like structures with faint or no staining. Extensive hemorrhage is frequently observed in cFUS-treated tissue (A), while rarely and minimally noted in pFUS-treated tissue. Scale bars represent 50 µm.
(TIF)

Figure S2 Expression of proinflammatory cytokines in muscle following cFUS or pFUS. Levels of each cytokine in treated muscle were not statistically different compared to the control tissue of the same day. ANOVA analyses did reveal that expression of IL-4, IL-5, and IL-17 in pFUS-treated tissue was elevated on days 0 and 1 compared to pFUS-treated tissue on days 3 and 7 even though no differences were observed between treated and control tissue on days 0 and 1. This finding may suggest a systemic increase of these cytokines on days 0 and 1 in response to pFUS exposures.
(TIF)

Table S1 Statistical analysis of cytokine array data following cFUS or pFUS.
(DOCX)

Table S2 Statistical analysis of expression of growth factors following pFUS.
(DOCX)
