# Supplementary figures and images for: Investigation of Cellular and Molecular Responses to Pulsed Focused Ultrasound in a Mouse Model
Source: PLoS One. 2011 Sep 13;6(9):e24730. doi: 10.1371/journal.pone.0024730 (PMC3172304; doi:10.1371/journal.pone.0024730)

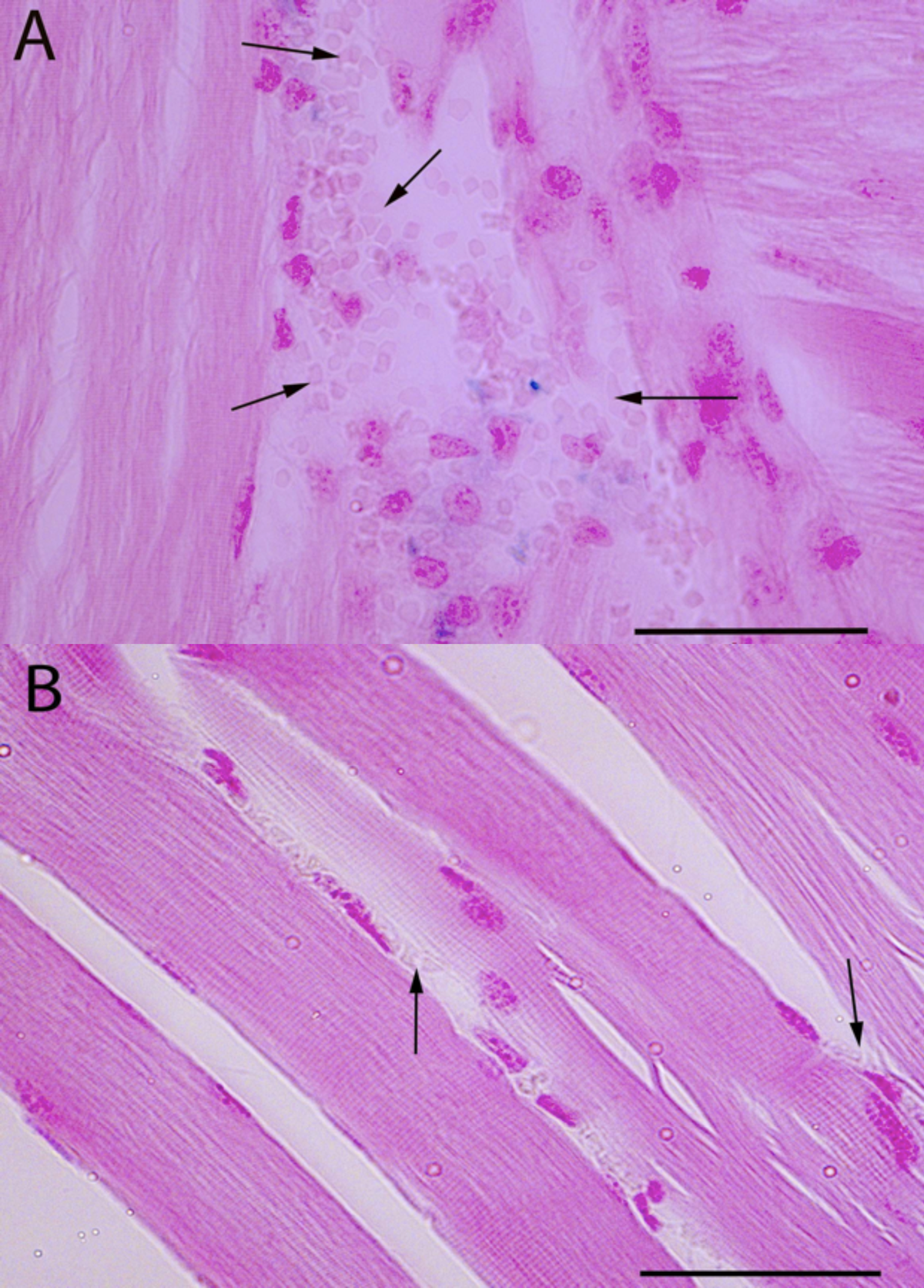

Supplement: Figure S1 — Prussian blue staining of FL-SPION-labeled macrophages in muscle tissue following FUS exposures. Muscle after cFUS (A) and pFUS (B) 3 days post-treatment. Using Nuclear Fast Red as a counterstain, anuclear red blood cells are indicated by arrows and appear as small disc-like structures with faint or no staining. Extensive hemorrhage is frequently observed in cFUS-treated tissue (A), while rarely and minimally noted in pFUS-treated tissue. Scale bars represent 50 µm. (TIF) [file pone.0024730.s001.tif]

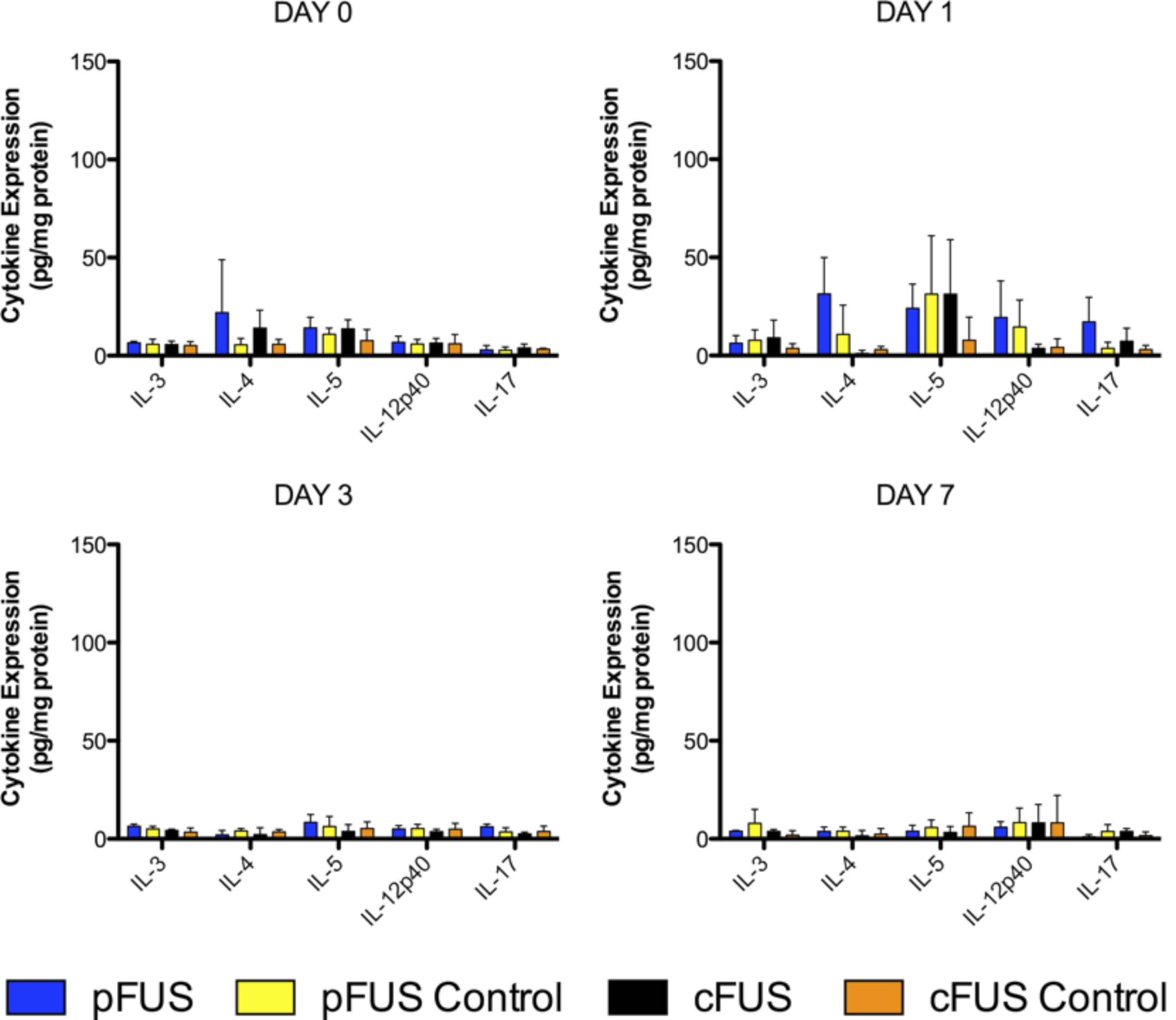

Supplement: Figure S2 — Expression of proinflammatory cytokines in muscle following cFUS or pFUS. Levels of each cytokine in treated muscle were not statistically different compared to the control tissue of the same day. ANOVA analyses did reveal that expression of IL-4, IL-5, and IL-17 in pFUS-treated tissue was elevated on days 0 and 1 compared to pFUS-treated tissue on days 3 and 7 even though no differences were observed between treated and control tissue on days 0 and 1. This finding may suggest a systemic increase of these cytokines on days 0 and 1 in response to pFUS exposures. (TIF) [file pone.0024730.s002.tif]
