# Supplementary material for: Investigation of Cellular and Molecular Responses to Pulsed Focused Ultrasound in a Mouse Model
Source: PLoS One. 2011 Sep 13;6(9):e24730. doi: 10.1371/journal.pone.0024730 (PMC3172304; doi:10.1371/journal.pone.0024730)
Supplement: Table S1 — Statistical analysis of cytokine array data following cFUS or pFUS. (DOCX) [file pone.0024730.s003.docx]

| **Table S1. Statistical analysis of cytokine array data following cFUS or pFUS** | | | | | | | | | | | |
| --- | --- | --- | --- | --- | --- | --- | --- | --- | --- | --- | --- |
|  | Two-way ANOVA | | | pFUS | | | | cFUS | | | |
|  |  |  |  | Bonferroni post test *p* values (treated vs. control) | | | | Bonferroni post test *p* values  (treated vs. control) | | | |
|  |  | *F* value  (*df in parentheses*) | *p* value | Day 0 | Day 1 | Day 3 | Day 7 | Day 0 | Day 1 | Day 3 | Day 7 |
| **IL-1α** | Interaction | (9, 64) = 10.81 | <0.0001 | NS | 0.0013 | 0.0013 | NS | NS | NS | NS | NS |
|  | Treatment | (3, 64) = 13.71 | <0.0001 |  |  |  |  |  |  |  |  |
|  | Time | (3, 64) = 7.50 | 0.0002 |  |  |  |  |  |  |  |  |
| **IL-1β** | Interaction | (9, 64) = 16.12 | <0.0001 | NS | 0.0009 | NS | NS | NS | NS | NS | NS |
|  | Time | (3, 64) = 23.97 | <0.0001 |  |  |  |  |  |  |  |  |
|  | Treatment | (3, 64) = 18.43 | <0.0001 |  |  |  |  |  |  |  |  |
| **IL-2** | Interaction | (9, 64) = 9.308 | <0.0001 | NS | NS | NS | NS | 0.0277 | NS | NS | NS |
|  | Time | (3, 64) = 8.940 | <0.0001 |  |  |  |  |  |  |  |  |
|  | Treatment | (3, 64) = 12.81 | <0.0001 |  |  |  |  |  |  |  |  |
| **IL-6** | Interaction | (9, 64) = 12.40 | <0.0001 | NS | NS | NS | NS | 0.0023 | 0.0139 | NS | NS |
|  | Time | (3, 64) = 16.11 | <0.0001 |  |  |  |  |  |  |  |  |
|  | Treatment | (3, 64) = 31.88 | <0.0001 |  |  |  |  |  |  |  |  |
| **IL-10** | Interaction | (9, 64) = 1.789 | 0.0876 | NS | NS | NS | NS | 0.0170 | NS | NS | NS |
|  | Time | (3, 64) = 2.766 | 0.0489 |  |  |  |  |  |  |  |  |
|  | Treatment | (3, 64) = 8.511 | <0.0001 |  |  |  |  |  |  |  |  |
| **MCP-1** | Interaction | (9, 64) = 10.81 | <0.0001 | 0.0147 | 0.0055 | NS | NS | 0.0007 | NS | NS | NS |
|  | Time | (3, 64) = 13.71 | 0.0002 |  |  |  |  |  |  |  |  |
|  | Treatment | (3, 64) = 7.50 | <0.0001 |  |  |  |  |  |  |  |  |
| **INFγ** | Interaction | (9, 64) = 18.38 | <0.0001 | NS | 0.0055 | NS | NS | NS | 0.04475 | NS | NS |
|  | Time | (3, 64) = 16.34 | <0.0001 |  |  |  |  |  |  |  |  |
|  | Treatment | (3, 64) = 18.82 | <0.0001 |  |  |  |  |  |  |  |  |
| **TNFα** | Interaction | (9, 64) = 1.410 | 0.2028 | 0.0153 | NS | NS | NS | NS | NS | NS | NS |
|  | Time | (3, 64) = 1.105 | 0.3536 |  |  |  |  |  |  |  |  |
|  | Treatment | (3, 64) = 0.3067 | 0.8204 |  |  |  |  |  |  |  |  |
| **MIP-1α** | Interaction | (9, 64) = 19.68 | <0.0001 | 0.0414 | 0.0005 | NS | NS | NS | NS | NS | NS |
|  | Time | (3, 64) = 21.79 | <0.0001 |  |  |  |  |  |  |  |  |
|  | Treatment | (3, 64) = 17.79 | <0.0001 |  |  |  |  |  |  |  |  |
| **GMCSF** | Interaction | (9, 64) = 11.44 | <0.0001 | NS | 0.0108 | NS | NS | NS | NS | NS | NS |
|  | Time | (3, 64) = 12.91 | <0.0001 |  |  |  |  |  |  |  |  |
|  | Treatment | (3, 64) = 10.98 | <0.0001 |  |  |  |  |  |  |  |  |
| Data analyzed by a two-way ANOVA treated time and treatment condition as independent variables. NS indicates “not significant” and a corrected *p* value >0.05 | | | | | | | | | | | |
