# Supplementary material for: Investigation of Cellular and Molecular Responses to Pulsed Focused Ultrasound in a Mouse Model
Source: PLoS One. 2011 Sep 13;6(9):e24730. doi: 10.1371/journal.pone.0024730 (PMC3172304; doi:10.1371/journal.pone.0024730)
Supplement: Table S2 — Statistical analysis of expression of growth factors following pFUS. (DOCX) [file pone.0024730.s004.docx]

| **Table S2. Statistical analysis of expression of growth factors following pFUS** | | |
| --- | --- | --- |
|  | *p* values (treated vs. control) | |
|  | Day 0 | Day 1 |
| **FGF** | 0.0083 | 0.0052 |
| **HGF** | 0.0123 | NS |
| **PDGF** | NS | NS |
| **PLGF** | 0.0373 | 0.0276 |
| **VEGF** | NS | 0.0054 |
| **SDF-1α** | 0.0131 | 0.0594 |
| *p* values are calculated using Student’s t-test with Bonferroni correction for multiple comparisons. NS indicates “not significant” and a corrected *p* value >0.05 | | |
